# Embolização arterial superseletiva para tratamento de angiomiolipoma em paciente com rim único

**DOI:** 10.1590/1677-5449.005515

**Published:** 2016

**Authors:** Adenauer Marinho de Oliveira Góes, Salim Abdon Haber Jeha, José Rui Couto Salgado

**Affiliations:** 1 Universidade Federal do Pará – UFPA, Belém, PA, Brasil.; 2 Centro Universitário do Estado do Pará – CESUPA, Belém, PA, Brasil.; 3 Hospital Porto Dias, Belém, PA, Brasil.

**Keywords:** angiomiolipoma, rim, embolização terapêutica

## Abstract

Os autores relatam o caso de uma paciente jovem previamente submetida a nefrectomia direita por apresentar angiomiolipomas renais (AMLRs) e portadora de dois volumosos angiomiolipomas no rim esquerdo remanescente. A paciente foi encaminhada pelo urologista para tratamento endovascular. Realizou-se embolização superseletiva de um dos tumores, localizado no polo renal inferior e em situação subcapsular; apesar de várias tentativas, não foi obtido um cateterismo seletivo suficiente para embolizar o segundo angiomiolipoma (localizado no polo renal superior) sem que um volume considerável de parênquima renal adjacente sofresse isquemia. O procedimento e a recuperação da paciente transcorreram sem complicações. A paciente recebeu alta no primeiro pós-operatório e vem sendo acompanhada ambulatorialmente há 9 meses sem intercorrências. É feita uma breve revisão sobre indicações, aspectos técnicos e complicações do tratamento endovascular dos AMLRs, além de serem discutidas vantagens dessa técnica quando comparada à ressecção cirúrgica dos tumores.

## INTRODUÇÃO

Angiomiolipomas renais (AMLRs) são tumores benignos hipervasculares que acarretam riscos de complicações hemorrágicas. São hamartomas compostos, em proporções variadas, por tecido adiposo e muscular e por vasos sanguíneos^1^^-^ 4. Trata-se de um tumor raro^1^^,^ 3 que representa 1% das massas renais, tem incidência de 0,07 a 0,3% da população^1^ e é duas vezes mais comum em mulheres^2^. Ocorre esporadicamente em 80^1^ a 90%^2^ dos pacientes, e em 10^2^ a 20% dos casos está associado a esclerose tuberosa complexa.

A remoção cirúrgica e a embolização são as opções de tratamento para AMLRs sintomáticos^3^. Atualmente, a embolização é o método de escolha pelo seu caráter minimamente invasivo, por previnir a ruptura tumoral em longo prazo, por ser capaz de preservar o parênquima renal normal adjacente ao tumor^1^^-^ 3 ^,^ 5 ^-^ 8 e por apresentar baixa ocorrência de complicações^1^^,^ 4 ^,^ 6 ^,^ 7.

O papel da embolização na vigência do sangramento já está bem estabelecido; entretanto, não há um consenso sobre quando intervir preventivamente. Os critérios habitualmente usados para indicar a embolização são tumores maiores que 4 cm, aneurismas intratumorais maiores que 4-5 mm, antecedente de sangramento por AMLR e ocorrência de dor abdominal/lombar^1^^,^ 2.

## PARTE I: RELATO DO CASO

Paciente de 25 anos, sexo feminino, portadora de esclerose tuberosa complexa, submetida a nefrectomia direita há 10 anos por angiomiolipomas renais e em acompanhamento urológico pela presença de angiomiolipomas no rim esquerdo.

**Figura 1:**
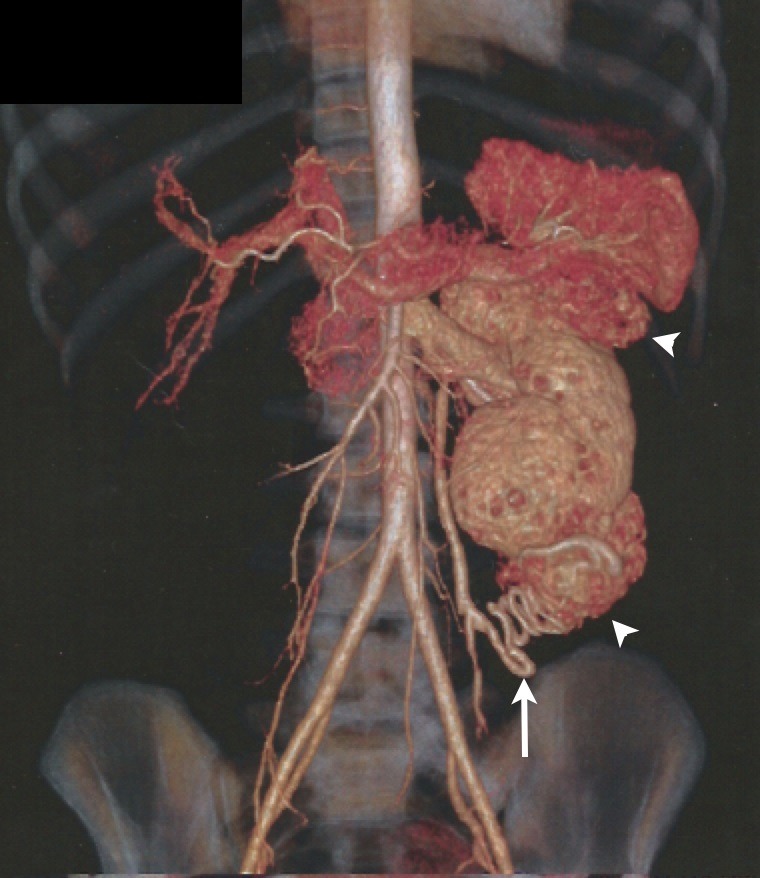
Reconstrução da tomografia computadorizada para planejamento do tratamento endovascular. As pontas das setas apontam os dois maiores tumores, localizados nos polos renais superior e inferior; a seta aponta o pedículo arterial associado a vascularização do tumor localizado no polo inferior. Notar a ausência do rim direito pela nefrectomia prévia.

Foi encaminhada para tratamento endovascular por apresentar três AMLRs no rim esquerdo, um no terço médio medindo 1,6 cm de diâmetro e dois tumores subcapsulares com prolongamentos exorrenais nos polos renais superior e inferior com diâmetros de 4,3 e 5,4 cm, respectivamente (Figura 1). A indicação do tratamento foi baseada no fato de a paciente ter rim único e nos tamanhos e localizações dos AMLRs. Exames laboratorias pré-operatórios, inclusive dosagens de ureia e creatinina, encontavam-se dentro de valores normais.

Foi planejada uma embolização superseletiva das artérias nutridoras dos tumores localizados nos polos renais superior e inferior.

## PARTE II: O QUE FOI FEITO

O procedimento foi realizado sob anestesia local e sedação, através de punção retrógrada da artéria femoral comum direita e implantação de um introdutor angiográfico 5F. Cateteres *pig-tail* e cobra curva 2 5F foram usados para as angiografias a partir da aorta abdominal e da artéria renal esquerda. As angiografias evidenciaram que o rim esquerdo era vascularizado por uma única artéria renal e por tumorações hipervascularizadas com extensão exorrenal localizadas nos polos renais superior e inferior cujo aspecto angiográfico era compatível com AMLRs.

**Figura 2:**
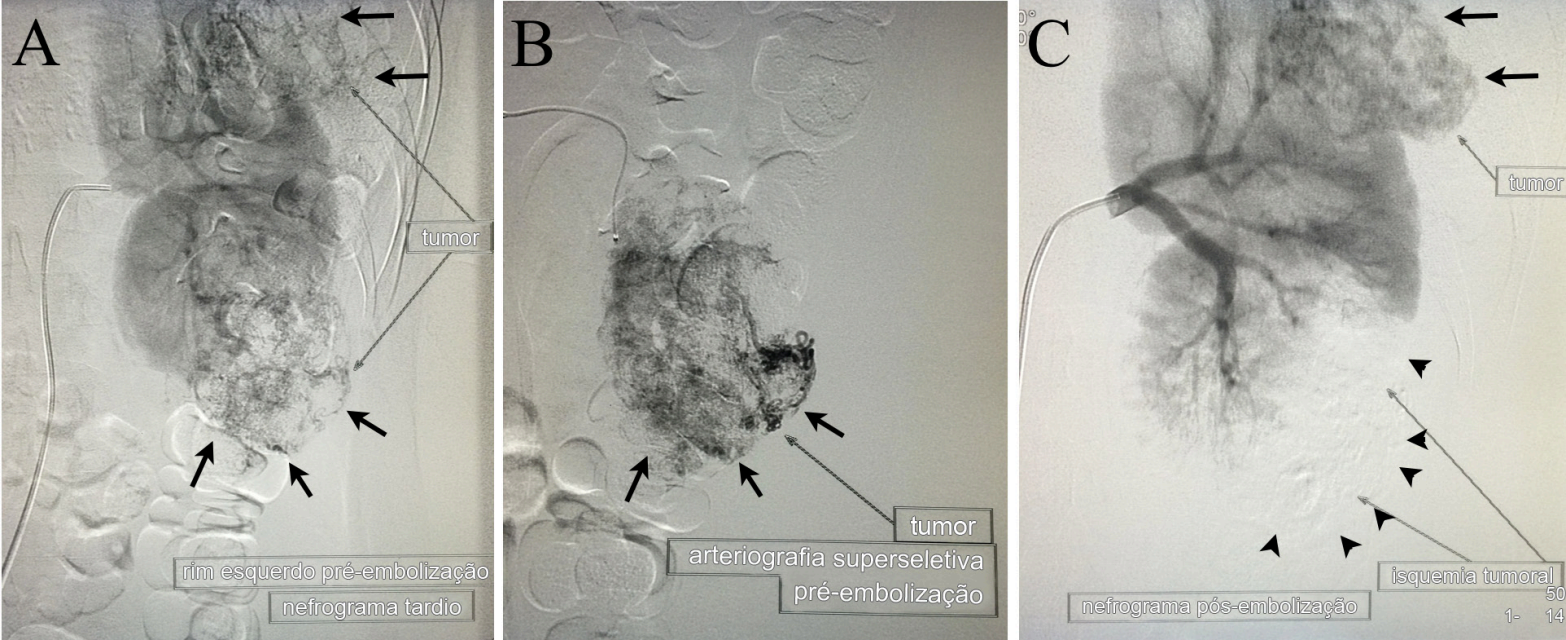
(A) Arteriografia renal seletiva pré-embolização; as setas apontam os tumores-alvo para a embolização. (B) Arteriografia superseletiva do pedículo nutridor do tumor localizado no polo renal inferior. (C) Arteriografia renal seletiva pós-embolização; as pontas das setas apontam a projeção do tumor no polo renal inferior (já embolizado), e as setas apontam o tumor localizado no polo renal superior (não embolizado); observar a preservação do parênquima renal adjacente ao tumor embolizado.

As angiografias superseletivas com microcateter Rebar (Covidien®) e microguia X-pedition (Meditronic®) demonstraram a hipervascularização displásica dos tumores. Os pedículos arteriais identificados como responsáveis pela irrigação do tumor no polo renal inferior foram embolizados com microesferas do tipo emboesferas (Medical®) de 300 a 500 µm. Apesar de várias tentativas, não foi obtido um cateterismo seletivo suficiente para embolizar o tumor subcapsular no polo superior sem que um volume considerável de parênquima renal adjacente sofresse isquemia. Optou-se pela interrupcão do procedimento. A angiografia de controle demonstrou importante diminuição da vascularização do AMLR do polo inferior (Figura 2).

A paciente retornou à enfermaria para suporte pós-operatório; não houve intercorrências dignas de nota, e ela recebeu alta no primeiro pós-operatório.

Após 9 meses de acompanhamento ambulatorial, não houve piora da função renal em relação ao pré-operatório, e a paciente segue sem queixas e sem indícios de complicações associadas ao AMLR não embolizado.

## DISCUSSÃO

A embolização da artéria renal foi descrita inicialmente em 1969 por Lalli e Peterson, principalmente no tratamento da hematúria e na paliação de tumores malignos do rim. Com o desenvolvimento dos materiais e o aumento da experiência com procedimentos endovasculares, as indicações passaram a englobar um vasto espectro de condições, como angiomiolipomas, malformações vasculares, embolizações pré-operatórias visando diminuição do sangramento intraoperatório^2^^,^ 6, sangramentos por lesões iatrogênicas (nefrolitotomia percutânea, biópsia, nefrostomia), ruptura de massas renais e traumas renais penetrantes e contusos^7^^,^ 8. A disponibilidade de cateteres de baixo perfil e agentes embólicos mais precisos diminuiu drasticamente a morbidade associada ao procedimento^6^.

A maioria dos AMLRs é diagnosticada incidentalmente^2^^,^ 3, pois 60% são assintomáticos; embora a taxa de crescimento do tumor seja imprevisível, ele tende a ocorrer. Há uma correlação entre o tamanho do AMLR e o surgimento de complicações e/ou sintomas^2^^,^ 3. A manifestação mais frequente é dor abdominal ou lombar em 85% dos casos, massa palpável em 53% e anemia em 21%. Sangramento retroperitoneal ou hematúria podem ocorrer, e a invasão do parênquima renal pode levar a insuficiência renal. Uma manifestação rara, já descrita, é a embolia pulmonar secundária à invasão da cava inferior pelo AMLR^9^.

Ultrassonografia (USG), tomografia computadorizada (TC) ou ressonância nuclear magnética (RNM) normalmente são suficientes para o diagnóstico, pois podem mostrar tecido adiposo no interior da massa renal. Calcificações, típicas de tumores mais agressivos, são raras nos AMLRs. Nesses casos, a RNM permite o diagnóstico diferencial. O carcinoma de células renais apresenta sinal de baixa intensidade em T1 e alta intensidade em T2, enquanto ocorre o oposto nos tecidos gordurosos^2^.

Além disso, na presença de sangramento, os AMLRs devem ser considerados um diagnóstico diferencial entre as massas renais, mesmo que não haja evidência de tecido gorduroso intralesional, pois sua presença pode ser mascarada pela hemorragia tumoral^2^.

Diante do aspecto radiológico característico, a indicação de biópsia é uma exceção, pois, por tratar-se de massa hipervascularizada, pode provocar hemorragia, e a possibilidade de seu resultado alterar a conduta terapêutica é mínima.

A angiografia evidencia vascularização anômala, com neovasos e microaneurismas. Os vasos são mais susceptíveis a aneurismas e ruptura por possuírem parede vascular pobre em tecido elástico normal e por apresentarem camada muscular substituída por tecido fibroso denso, o que justifica a predisposição do tumor a hemorragias^2^.

Entre os critérios de intervenção, encontram-se: diâmetro maior que 4 cm (para alguns autores, 3,5 cm), aneurismas intratumorais maiores que 4-5 mm^1^^,^ 2 e a ocorrência de dor, hemorragia ativa^1^, AMLRs múltiplos, bilaterais ou unilaterais em rim único e pacientes com esclerose tuberosa complexa^1^^,^ 2 ^,^ 7.

Vários estudos já demonstraram a eficácia da embolização no tratamento e prevenção de hemorragia^1^^-^ 4.

O procedimento pode ser feito sob anestesia local, com ou sem sedação; porém, alguns autores acreditam que o procedimento pode ser executado de forma mais rápida e segura sob anestesia geral^6^. O procedimento pode ser demorado quando são necessárias várias tentativas até se obter um cateterismo superseletivo; nessas condições adversas, a imobilidade e a apneia do paciente facilitam o procedimento.

Uma aortografia completa deve preceder a embolização para avaliar a existência de artérias renais acessórias ou outras artérias associadas à vascularização do tumor^6^. A embolização superseletiva pode promover uma oclusão controlada de minúsculos ramos arteriais que irrigam a lesão com mínimo comprometimento da vascularização do parênquima normal adjacente à lesão^3^^,^ 6 ^-^ 8. Além disso, frequentemente, possibilita a preservação de mais néfrons funcionantes do que a ressecção cirúrgica da lesão^3^^,^ 7. No caso relatado, fatores como o prolongamento da duração do procedimento, a necessidade de injeções adicionais de contraste iodado e a possibilidade de isquemia de parênquima renal pela não obtenção de cateterismo superseletivo em uma paciente com rim único contribuíram para a decisão de interromper o procedimento sem que a embolização do tumor localizado no polo renal superior fosse realizada. Com base no diâmetro da lesão, é provável que uma nova tentativa de embolização direcionada especificamente para essa lesão seja realizada, dependendo da indicação do urologista assistente.

Diferentes agentes embólicos já foram descritos na literatura para o tratamento de AMLRs, como partículas de polivinil álcool (PVA), etanol, microesferas, *gelfoam*, molas^1^^-^ 3 ^,^ 6 ^,^ 8, lipiodol^2^^,^ 6, cola de n-butil-cianoacrilato, sotradecol^6^ e ônix^2^.

O PVA possui a desvantagem de apresentar partículas de tamanho e contorno irregulares, predispondo obstrução do microcateter; a falta de homogeneidade das partículas também pode acarretar penetração insatisfatória do agente nas porções mais distais dos vasos tumorais^2^.

As microesferas calibradas são de fácil manipulação; sua diluição no contraste iodado e a utilização dos recursos de *zoom* durante a injeção permitem o acompanhamento do agente embolizante e, por apresentarem superfície e tamanho regulares, raramente obstruem o microcateter. Por essas características, esse foi o material escolhido para este caso^2^.

O uso de molas deve ser criterioso, pois, uma vez liberadas, impedem o acesso aos segmentos mais distais do vaso no qual elas foram implantadas, o que pode ser necessário em reintervenções precoces ou tardias. Já foram relatadas rupturas de aneurismas em AMLRs após a embolização com molas de segmentos distais dos vasos em que esses aneurismas se encontravam; a teoria proposta é de que, ao se ocluir o vaso distalmente ao aneurisma, haveria um aumento da pressão sobre suas paredes, levando à sua ruptura. O implante de molas dentro do aneurisma ou em situação proximal pode ser feito com o intuito de prevenir sua ruptura^2^.

Embora diretrizes recentes recomendem o uso de microesferas com diâmetro maior que 500 µm para evitar a passagem do material através de comunicações arteriovenosas intratumorais^1^, ainda não há consenso na literatura sobre a superioridade de um agente embolizante específico no tratamento do AMLR. A escolha deve considerar a familiaridade do médico com o agente e a disponibilidade do material^2^^,^ 3.

As complicações após a embolização preventiva de AMLR são raras^1^^,^ 4. A mais frequente é a síndrome pós-embolização (SPE), caracterizada por dor, febre, náuseas e vômitos autolimitados nos primeiros dias após a embolização^1^^,^ 4 ^,^ 8 ^,^ 10. A ocorrência de SPE varia de cerca de 39^10^ a 63%^2^ em séries de casos previamente publicadas. A formação de abscesso renal pode acometer cerca de 5%, e o derrame pleural, 3%; hematoma no sítio de punção^2^ e migração do agente embolizante com isquemia de outros órgãos^8^, embora raros, também podem ocorrer.

Pode acontecer necrose liquefativa do tecido adiposo que constitui o tumor^1^^,^ 2 ^,^ 4 em até 20% dos pacientes, principalmente em AMLRs com maior conteúdo adiposo (mais que 50% da massa tumoral); ao contrário da SPE, habitualmente manifesta-se meses após a embolização^1^ através de dor lombar, febre e/ou lipidúria^2^. Outras complicações tardias incluem abscesso renal ou perirrenal, perda de função renal, hipertensão renovascular^10^ e fístulas renoduodenal e renocolônica^4^.

A principal vantagem da embolização sobre a ressecção tumoral é a preservação do parênquima renal funcionante^2^. Além disso, destacam-se o caráter minimamente invasivo do procedimento endovascular, a baixa ocorrência de complicações precoces e tardias, a possibilidade de ser realizado sob anestesia local e o mínimo sangramento intraoperatório^7^.

Em casos de hemorragias ativas, o procedimento apresenta taxas de sucesso de até 86%, além de levar à redução gradual do tumor. Eletivamente, ele previne hemorragias em até 94%, e a duração da internação hospitalar não costuma exceder 24 horas^2^.

No pós-operatório, a redução do tumor não deve ser utilizada como parâmetro isolado ao se avaliar o sucesso da embolização. Devem ser considerados o desaparecimento de sintomas inicialmente presentes, a ausência de crescimento do tumor e a não recorrência de hemorragias^2^.
