# Supplementary material for: Embolização arterial superseletiva para tratamento de angiomiolipoma em paciente com rim único
Source: J Vasc Bras. 2016 Apr-Jun;15(2):142–6. [Article in Portuguese] doi: 10.1590/1677-5449.005515 (PMC5829709; doi:10.1590/1677-5449.005515)
Supplement: Translation

# Translation: Superselective arterial embolization for treatment of angiomyolipoma in a patient with a single kidney

**Authors:** Adenauer Marinho de Oliveira Góes, Salim Abdon Haber Jeha, José Rui Couto Salgado

**Affiliations:** 1 Universidade Federal do Pará – UFPA, Belém, PA, Brazil.; 2 Centro Universitário do Estado do Pará – CESUPA, Belém, PA, Brazil.; 3 Hospital Porto Dias, Belém, PA, Brazil.

**Keywords:** angiomyolipomas, kidney, therapeutic embolization

## Abstract

The authors report on a case of a young woman who had previously undergone a right nephrectomy due to renal angiomyolipomas, currently presenting voluminous angiomyolipomas of the remaining kidney. The patient’s urologist referred her for endovascular treatment. Superselective arterial embolization of one tumor (located at the inferior renal pole), was conducted successfully. Several attempts at selective catheterization were made to embolize the second angiomyolipoma (located at the superior lobe), without jeopardizing a significant amount of the surrounding renal parenchyma, but this ultimately proved not to be feasible. The procedure and recovery were uneventful. The patient was discharged on the first postoperative day and has been followed for 9 months with no complications. The authors provide a brief review of the indications, technical aspects and complications of endovascular treatment of renal angiomyolipomas and also discuss the advantages of the endovascular approach over surgical resection for this kind of tumor.

## INTRODUCTION

Renal angiomyolipomas (RAMLs) are benign hypervascular tumors that involve a risk of hemorrhagic complications. They are hamartomas and they are composed, in varying proportions, of adipose and muscle tissues and blood vessels.^1^^-^ 4 They are rare tumors^1^^,^ 3 that account for 1% of renal masses, they have an incidence of 0.07 to 0.3% of the population,^1^ and they are twice as common among women.^2^ They occur sporadically in 80^1^ to 90%^2^ of patients, while in 10^2^ to 20% of cases they are found in combination with tuberous sclerosis complex.

The treatment options for symptomatic RAMLs are surgical removal or embolization.^3^ Nowadays, embolization is the method of choice because of its minimally invasive character, because it prevents tumour rupture over the long term, because it has the capacity to preserve the normal renal parenchyma adjacent to the tumor,^1^^-^ 3 ^,^ 5 ^-^ 8 and because it is associated with a low rate of complications.^1^^,^ 4 ^,^ 6 ^,^ 7

The role of embolization in the presence of bleeding is already well-established. However, there is no consensus on when to intervene preventively. The criteria habitually used to indicate embolization are tumor size greater than 4 cm, intratumoral aneurysms larger than 4-5 mm, history of bleeding due to RAML and ocurrence of abdominal/lumbar pains.^1^^,^ 2

## PART I: CASE REPORT

The patient was a 25-year-old female with tuberous sclerosis complex, who had undergone a right nephrectomy 10 years previously because of renal angiomyolipomas and was being monitored by a urologist because of the presence of angiomyolipomas in the left kidney.

She was referred for endovascular treatment because three RAMLs had been detected in the left kidney, one in the mid third measuring 1.6 cm in diameter and two subcapsular tumors at the upper and lower poles of the kidney with extrarenal projections and diameters of 4.3 and 5.4 cm, respectively (Figure 1). The decision to treat was based on the fact that the patient only had one kidney and on the sizes and sites of the RAMLs. Preoperative laboratory test results, including urea and creatinine assays, were within normal limits.

**Figure 1:** Reconstruction from the computed tomography executed for planning of endovascular treatment. The arrowheads indicate the two larger tumors, located at the upper and lower poles of the kidney; the arrow indicates the arterial pedicle associated with vascularization of the lower pole tumor. Note the absence of the right kidney due to the previous nephrectomy.

Superselective embolization of the arteries feeding the tumors at the upper and lower poles of the kidneay was planned.

## PART II: WHAT WAS DONE

The procedure was conducted under local anesthesia and sedation, achieved via retrograde puncture of the right common femoral artery and placement of a 5F angiographic introducer. Pig-tail and curved cobra 2 5F catheters were used to conduct angiographs, via the abdominal aorta and the left renal artery. The angiographs showed that the left kidney was vascularized by a single renal artery and by hypervascularized tumors with extra-renal extensions located at the upper and lower poles of the kidney and with an angiographic appearance compatible with RAMLs.

Superselective angiographs using a Rebar microcatheter (Covidien®) and X-pedition microguide (Meditronic®) showed the tumors’ dysplastic hypervascularization. The arterial pedicles identified as responsible for feeding the tumor in the lower renal pole were embolized using 300 to 500 µm embosphere type microspheres (Medical®). Although several attempts were made, it did n ot prove possible to achieve catheterization that was sufficiently selective to embolize the subcapsular tumor in the upper pole without subjecting a considerable volume of the adjacent renal parenchyma to ischemia. A decision to abort the procedure was taken. A control angiography showed considerable reduction in vascularization of the RAML in the lower pole (Figure 2).

**Figure 2:** (A) Selective arteriography of the kidney prior to embolization; the arrows indicate the target tumors for embolization. (B) Superselective arteriography of the pedicle feeding the tumor at the lower pole of the kidney. (C) Selective renal arteriography after embolization; the arrowheads indicate the projection of the lower pole tumor (already embolized), and the arrows indicate the upper pole tumor (which was not embolized); observe the preserved renal parenchyma adjacent to the embolized tumor.

The patient retrurned to the ward for postoperative support, there were no notable intercurrent conditions, and she was discharged on the first postoperative day.

After 9 months’ outpatients follow-up, there has been no deterioration in renal function with relation to the patient’s preoperative status and she is free from complaints or signs of complications associated with the RAML that could not be embolized.

## DISCUSSION

Embolization of the renal artery was first described in 1969 by Lalli and Peterson, primarily for treatment of hematuria and as paliative treatment for malignant kidney tumors. As materials have evolved and experience has been accrued with endovascular procedures, indications have come to include a vast spectrum of conditions including angiomyolipomas, vascular malformations, preoperative embolizations with the objective of attenuating intraoperative bleeding,^2^^,^ 6 bleeding caused by iatrogenic injuries (percutaneous nephrolithotomy, biopsy, nephrostomy), ruptures of renal masses and penetrating and blunt renal traumas.^7^^,^ 8 The availability of low profile catheters and more precise embolic agents have dramatically reduced morbidity associated with the procedure.^6^

The majority of RAMLs are diagnosed incidentally,^2^^,^ 3 because 60% are asymptomatic; and while the tumoral growth rate is unpredictable, the tendency is that growth will occur. There is a correlation between RAML size and the occurrence of complications and/or symptoms.^2^^,^ 3 The most common manifestation is abdominal or lumbar pains in 85% of cases, palpable mass in 53% and anemia in 21%. Retroperitoneal bleeding or hematuria may also occur, and invasion of the renal parenchyma can lead to kidney failure. One rare manifestation that has been described is pulmonary embolism secondary to invasion of the inferior cava by the RAML.^9^

Ultrasonography (USG), computed tomography (CT) or magnetic resonance imaging (MRI) are normally sufficient for a diagnosis, since they can identify adipose tissue in the interior of the renal parenchyma. Calcifications typical of more aggressive tumors are rare in RAMLs. In these cases, MRI enables differential diagnosis. Renal cell carcinomas exhibit a low intensity signal on T1 and a high intensity signal on T2, whereas the opposite is true of fatty tissues.^2^

Additionally, in the presence of bleeding, RAMLs should be considered on the list of possible differential diagnoses of renal masses, even when there is no sign of fatty tissues within the lesion, because they could be masked by tumoral hemorrhage.^2^

If X-ray findings are characteristic of an RAML, its hypervascularized nature means that biopsy is only indicated in exceptional circumstances because of the risk of hemorrhage and since there is a minimal possibility that the results will change the therapeutic management chosen.

Angiography will show anomalous vascularization, with neovessels and microaneurysms. These vessels are more susceptible to aneurysm and rupture because the vascular wall has a lower normal elastic tissue content and the layer of muscle tissues is substituted by dense fibrous tissue, which explains these tumors’ predisposition to hemorrhage.^2^

Criteria for intervention include diameter greater than 4 cm (some authors state 3.5 cm), intratumoral aneurysms larger than 4-5 mm^1^^,^ 2 and pain, active hemorrhage,^1^ multiple RAMLs, bilateral RAMLs or unilateral RAML when the patient only has one kidney, and patients with tuberous sclerosis complex.^1^^,^ 2 ^,^ 7

Several different studies have demonstrated the efficacy of embolization for treatment and prevention of hemorrhage.^1^^-^ 4

The procedure can be performed under local anesthesia, with or without sedation, but some authors believe that the procedure can be conducted more quickly and with greater safety under general anesthesia.^6^ The procedure may take a considerable time when several attempts are needed to achieve superselective catheterization and under these adverse conditions an immobile patient in a state of apnea facilitates the procedure.

A full aortography should be conducted in advance of embolization in order to assess the presence of accessory renal arteries or other arteries associated with vascularization of the tumor.^6^ Superselective embolization can provoke controlled occlusion of miniscule arterial branches that feed the tumor, with minimum compromise to vascularization of the adjacent normal parenchyma.^3^^,^ 6 ^-^ 8 Furthermore, it will often enable preservation of more functional nephrons than surgical resection.^3^^,^ 7 In the case described above, factors such as extending the duration of the procedure, the need for additional injections of iodinated contrast and the possibility of ischemia of the renal parenchyma because of failure to obtain superselective catheterization in a patient with only one kidney all contributed to the decision to end the procedure without having achieved embolization of the tumor located at the upper pole of the kidney. Considering the diameter of the tumor, it is likely that another attempt will be made at embolization directed specifically at this tumor, depending on the opinion of the treating urologist.

Several different embolic agents for treatment of RAMLs have been described in the literature, including particles of polyvinyl alcohol (PVA), ethanol, microspheres, gelfoam, coils,^1^^-^ 3 ^,^ 6 ^,^ 8 lipiodol,^2^^,^ 6 n-butyl-cyanoacrylate adhesive, sotradecol^6^ and onyx.^2^

The disadvantage of PVA is that its particles have irregular size and shape, making obstruction of the microcatheter more likely, and the lack of particle uniformity can also cause unsatisfactory penetration of the agent into the more distal portions of the tumor vessels.^2^

Calibrated microspheres are easy to handle, diluting them in iodinated contrast and employing the zoom facility during injection makes it possible to monitor the embolization agent, and since they have regular sizes and surfaces, they rarely obstruct the microcatheter. It was because of these characteristics that this was the agent chosen in this case.^2^

Coils should be used with care because once released they block access to more distal segments of the vessel into which they are released and which could be needed for early or late reinterventions. Ruptures of aneurysms in RAMLs have been reported after coil embolization of distal segments of the vessels in which the aneurysms were located. The theory proposed to explain this is that occluding the vessel distal of the aneurysm increased the pressure on its walls, causing it to rupture. Coils can be released into the aneurysm or a proximal site, with the objective of preventing it from rupturing.^2^

Although recent guidelines recommend using microspheres with diameters larger than 500 µm, to avoid their passage through the intratumoral arteriovenous communications,^1^ there is not yet a consensus in the literature on the superiority of any one specific embolization agent for treatment of RAML. The choice of embolization agent should consider the physician’s familiarity and the materials available.^2^^,^ 3

Complications after preventative embolization of RAMLs are rare.^1^^,^ 4 The most frequent is post-embolization syndrome (PES), which is characterized by pain, fever, and self-limiting vomiting and nausea during the first days after embolization.^1^^,^ 4 ^,^ 8 ^,^ 10 The occurrence of PES varies from 39^10^ to 63%^2^ in published case series. Renal abscess formation can occur in around 5%, and pleural effusion in 3% of cases. Hematoma at the puncture site^2^ and migration of the embolization agent causing ischemia of other organs^8^ are rare, but can occur.

Liquefactive necrosis of the adipose tissue making up the tumor^1^^,^ 2 ^,^ 4 can occur in up to 20% of patients, primarily in RAMLs with a higher adipose content (more than 50% of the mass of the tumor) and, in contrast with PES, it habitually manifests months after embolization,^1^ with lumbar pain, fever and/or lipiduria.^2^ Other later complications include renal or perirenal abscess, loss of kidney function, renovascular hypertension,^10^ and renoduodenal and renocolonic fistulas.^4^

The principal advantage that embolization offers over resection of the tumor is preservation of the functioning renal parenchyma.^2^ Also important are the minimally invasive character of the endovascular procedure, the low rate of early and late complications, the possibility of being performed under local anesthesia, and minimal intraoperative bleeding.^7^

In cases with active hemorrhage, the procedure has success rates of up to 86%, in addition to provoking gradual reduction of the tumor. As an elective procedure it prevents hemorrhages in up to 94% of cases, and duration of hospital stay does not tend to exceed 24 hours.^2^

Postoperatively, reduction of the tumor should not be used as the only parameter for assessing the success of embolization. Disappearance of the symptoms that were initially present, absence of tumor growth and non-recurrence of hemorrhages should also be considered.^2^
